# Phase II Trial of Doxorubicin Plus Escalated High-Dose Ifosfamide in Patients With Advanced Soft Tissue Sarcomas of the Adult: A Study of the Spanish Group for Research on Sarcomas (GEIS)

**DOI:** 10.1155/SRCM/2006/26986

**Published:** 2006-09-19

**Authors:** A. López-Pousa, J. Martín, J. Montalar, R. de las Peñas, J. García del Muro, J. Cruz, J. Maurel, P. Escudero, A. Casado, J. M. Buesa, the Spanish Group for Research on Sarcomas (GEIS)

**Affiliations:** ^1^Medical Oncology Department, Hospital Sant Pau, 08025 Barcelona, Spain; ^2^Medical Oncology Department, Hospital Son Dureta, 07014 Palma de Mallorca, Spain; ^3^Medical Oncology Department, Hospital Clínico La Fe, 46009 Valencia, Spain; ^4^Medical Oncology Department, Hospital Provincial, 12002 Castellón, Spain; ^5^Medical Oncology Department, Instituto Catalán de Oncología, 08907 Barcelona, Spain; ^6^Medical Oncology Department, Hospital Universitario de Canarias, 38320 Tenerife, Spain; ^7^Medical Oncology Department, Hospital Clínic, 08036 Barcelona, Spain; ^8^Medical Oncology Department, Hospital Clínico, 50009 Zaragoza, Spain; ^9^Medical Oncology Department, Hospital Clínico San Carlos, 28040 Madrid, Spain; ^10^Medical Oncology Department, Hospital Central de Asturias, Instituto Universitario de Oncología del Principado de Asturias (IUOPA), 33006 Oviedo, Spain

## Abstract

*Background.* To explore the tolerance and the activity of high-dose ifosfamide (IFOS) combined with doxorubicin (DXR) at 50 mg/m^2^ every 4 weeks in patients with soft tissue sarcomas. *Methods.* DXR was given IV bolus and IFOS by continuous infusion at 2 g/m^2^/day. Initial IFOS dose (12 g/m^2^) was adjusted to 10, 13, or 14 g/m^2^ according to toxicity. *Results.* Seventy patients received 277 cycles (median 3 cycles, range 1–10), 34% with IFOS dose increased, 30% decreased, and 48% delivered at 12 g/m^2^. Toxicity grade 4 occurred on granulocytes (67% of patients) or platelets (19%), 54% had febrile neutropenia, 31% grade 3/4 asthenia, and 26% abandoned the study due to toxicity. Three toxic deaths occurred. In 57 non-GIST patients objective activity was 45.6% (95% CI, 32 to 58%). *Conclusion.* At least 4 cycles were tolerated by 71% of patients, most receiving DXR 50 mg/m^2^ plus IFOS 10–12 g/m^2^, with substantial toxicity.

## INTRODUCTION

Doxorubicin (DXR) and ifosfamide (IFOS) are two active agents against advanced soft tissue sarcomas (ASTS), which have been combined in an attempt to improve therapeutic efficacy [1]. However, the combination of DXR 50 mg/m^2^ plus IFOS 5 g/m^2^ was not more active than single agent DXR at 75 mg/m^2^ in an EORTC study [2], although other authors have reported a higher response rate when 7.5 g/m^2^ IFOS was added to DXR [3] or to a combination of DXR and dacarbazine [4]. The objective activity detected for those IFOS plus DXR combinations ranged from 25% to 34%. The concourse of hematopoietic growth factors allowed the administration of full DXR doses, but a comparative study of IFOS at 5 g/m^2^, combined with 50 or 75 mg/m^2^ DXR, was negative with respect to efficacy and overall survival, with progression-free survival being significantly longer in the high-dose arm [5].

Second-line studies conducted at higher IFOS doses disclosed a dose-response relationship for this agent [6, 7], what originated a series of single-arm Phase II trials combining an anthracycline with high-dose IFOS (≥ 10 g/m^2^). At the MD Anderson Hospital Cancer Center, two consecutive trials explored DXR doses of 75 or 90 mg/m^2^ added to 10 g/m^2^ IFOS. All patients included had at least one episode of febrile neutropenia, and thrombocytopenia was significant in both pilot trials, so that 50% of patients had toxicity grade 4 on platelets [8]. When IFOS 12.5 g/m^2^ was combined with DXR at 60 mg/m^2^, grade 4 thrombocytopenia occurred in 13% of patients, and 74% received 4 to 6 cycles at full doses [9]. In another study, the combination of 12.5 g/m^2^ IFOS with 4-epidoxorubicin (EPI) at 90 mg/m^2^ induced grade 4 thrombocytopenia in 35% of patients [10], while EPI at 110 mg/m^2^ plus IFOS 10 g/m^2^ were the doses recommended after a Phase I trial [11]. In those trials, grade 4 neutropenia occurred in 70–100% of patients, a toxicity that was not considered limiting. Objective activity of those regimens, usually delivered every 3 weeks, varied from 52% to 69%, although randomized studies comparing their efficacy with that of single agent DXR or standard-dose combinations are still lacking.

When this study was planned it appeared that, in those high-dose regimens, the dose of one of the agents should be compromised in order to cause a toxicity that would allow the administration of at least 4 to 6 cycles. Furthermore, our group had recently concluded a first-line trial with IFOS given at 14 g/m^2^ every 4 weeks [12]. With those antecedents, we decided to conduct a study to determine the dose of IFOS that would cause an acceptable toxicity when combined with 50 mg/m^2^ DXR. The initial dose of 12 g/m^2^ IFOS was adjusted in subsequent cycles in function of the hematologic toxicity encountered in each patient. Our purpose was also to evaluate the activity of the combination.

## MATERIALS AND METHODS

### Selection criteria

The criteria for inclusion in the trial were a histologic diagnosis of soft tissue sarcoma (malignant mesothelioma and extraosseous chondrosarcoma, osteosarcoma or Ewing/PNET were excluded), locally advanced or metastatic disease, age between 15 and 65 years, no prior chemotherapy either adjuvant or for advanced disease, bidimensionally measurable disease progressing in the last 4 weeks, performance status (WHO) ≤ 2, leukocytes > 4.000/mm^3^, platelets > 100.000/mm^3^, serum creatinine < 1.5 mg/dL, creatinine clearance > 60 mL/min, total bilirubin > 1.5 mg/dL, and albumin > 30 g/L. Patients had to have a left ventricle ejectional fraction (LVEF) at baseline > 50%. Patients were excluded if they had a severe associated disease, CNS metastases, a second primary tumor (except for adequately treated in situ cervical carcinoma and squamous or carcinoma of the skin), if they had received radiation therapy on the only measurable lesion, or if difficulties in follow-up were expected. The study protocol was approved by the Ethical Committee of each participating institution and an informed written consent was obtained from all patients.

### Treatment

All patients received doxorubicin at a fixed dose of 50 mg/m^2^ infused IV over 10–20 minutes on day 1 immediately followed by a continuous infusion of ifosfamide (IFOS) with GM-CSF support, every 4 weeks. The initial dose of IFOS was 12 g/m^2^ (plus 50% Mesna) (Pras-Pharma, Madrid, Spain) and was given according to the following schedule: on day 1 500 mL of normal saline (NS) were administered IV in 1 hour, followed by IFOS 2 g/m^2^ plus Mesna 1 g/m^2^ in 1.000 mL NS in 2 hours, and the rest of the dose was infused at a rate of 2 g/m^2^ IFOS every 24 hours. Daily dose was diluted in 1.000 mL NS. At the end of IFOS infusion 3 additional doses of Mesna 600 mg/m^2^ every 6 hours were given, either orally or IV, with an oral intake of 1.500 mL or 1.500 mL NS IV in 12 hours. All patients received sodium bicarbonate 1/6 M 250 mL/day (40 mEq/day) with IFOS infusion. No extra fluid intake was required during therapy, and patients were instructed to void frequently. If they noted dysuria, an additional dose of Mesna 1 g IV bolus was administered and the amount of Mesna in the rest of the infusion was increased to a 100% that of IFOS. Prophylactic GM-CSF (molgramostin, Schering-Plough SA, Barcelona, Spain) was started on day 7 and given subcutaneously at 5 *μ*g/Kg/day for 10 consecutive days, or until leukocyte count ≥ 10.000/mm^3^. Values of hemoglobin were kept above 10 g/100 mL transfusing red-packed cells when necessary.

Weekly BCC were performed, and cycles were repeated if leukocytes were ≥ 3.000/mm^3^, granulocytes were ≥ 1.500/mm^3^, and platelets were ≥ 100.000/mm^3^ on day 28. Otherwise, cycles were delayed until reaching those figures, and the patient interrupted therapy if no recovery was detected after 3 weeks. There were four dose levels of IFOS in this study (10, 12, 13, and 14 g/m^2^), while the dose of doxorubicin was kept constant at 50 mg/m^2^. IFOS dose was adapted to the toxicity observed in the prior cycle with the duration of the infusion modified to maintain the rate of 2 g/m^2^/24 h. The dose was progressively increased to 13 and 14 g/m^2^ if at nadir leukocytes were ≥ 1.000/mm^3^ and platelets were ≥ 75.000/mm^3^ in the absence of fever. The dose was reduced one level when neutropenic fever was observed, if platelets were < 50.000/mm^3^ for more than 1 week, if platelets were < 25.000/mm^3^, or if stomatitis grade 3 to 4 was reported. If any of those situations recurred at 10 g/m^2^, the patient was to abandon the trial. The patient was also withdrawn from the study if creatinine clearance was < 60 mL/L or serum creatinine was > 2.5 times than normal upper value, if a decrease in LVEF > 15% with respect to baseline was detected, or after any toxicity that might endanger the life of the patient. In the presence of transient hallucinations the patient continued on study, but no if important somnolence, confusion, toxic psychosis or coma (grade 3 to 4 neurocortical toxicity) occurred.

Antiemesis consisted of dexamethasone 8 mg IV bolus plus either granisetron 3 mg or ondansetron 8 mg IV on day 1, according to each center's routine, followed by oral granisetron 1 mg or ondansetron 4 mg every 12 hours for 6 consecutive days. The use of psychotropic drugs such as benzodiazepines and narcotic analgesics was discouraged during the infusion of IFOS, as well as the use of aminoglycosides to treat febrile episodes.

Baseline studies included a clinical history and physical examination, evaluation of the performance status, assessment of the different tumoral lesions and measurement of all evaluable lesions (physical examination, thorax X-ray, computed tomographic scan, but not ultrasound, were valid procedures), thorax X-ray, ECG, LVEF, complete and differential cell blood count (CBC), serum chemistries with glucose, sodium, potassium, calcium, magnesium, phosphorus, bicarbonate, BUN, creatinine, total protein, albumin, AST, ALT, alkaline phosphatase, LDH, total bilirubin, urine examination by microscopy and dipstick, and creatinine clearance. A complete CBC was performed at the end of IFOS infusion and weekly thereafter, while physical examination, evaluation of the performance status, CBC, blood chemistries and microscopic examination of the urine were repeated before each new cycle. A thorax X-ray and the measurement of all evaluable lesions were repeated every 8 weeks, and LVEF was determined every 4 cycles.

WHO criteria were followed to evaluate response to therapy [13]. Any objective remission had to be confirmed at least 4 weeks later and was externally reviewed. Complete remission (CR) was the complete disappearance of all signs and symptoms of disease for at least four weeks, and a pathologic complete response (pCR) was the absence of viable tumor in the surgical specimen of the evaluated lesion. Partial remission (PR) was considered a decrease of at least 50% in the sum of the products of the two longest perpendicular diameters of every measurable lesion, in the absence of any progressive lesion, and stable disease (SD) was any change that did not qualify for either a partial response or progressive disease. The appearance of a new lesion or an increase of at least 25% in any measurable lesion was termed progressive disease (PD), while rapid progression was the appearance of a new lesion or an increase in any measurable lesion higher than 50% after only one cycle of therapy. In the absence of rapid progression, at least 2 cycles were necessary to evaluate antitumor activity. Therapy continued in the presence of a PR or SD and the maximum number of cycles per protocol was 6, although rescue surgery or other potentially curative procedures could be attempted at the discretion of each investigator only after 4 cycles of therapy for reasons of uniformity. Patients could continue on protocol after 6 cycles at investigator discretion. All patients receiving at least 1 cycle were considered evaluable for toxicity, and those cycles with weekly CBC were assessable for hematologic toxicity. Toxicity was graded according to Common Toxicity Criteria of the National Cancer Institute (NCI) (Version 1.0). Dose-intensity per patient was calculated by dividing total dose administered per square meter by the total duration of treatment in weeks plus 4 additional weeks.

The duration of objective remissions was measured from the date first observed until the date of progression, and time to progression was measured from the date of inclusion until progressive disease was first observed. In both analysis patients were censored on the date any other therapeutic procedure (ie, rescue surgery) was performed. Overall survival was measured from the date of patient inclusion until death from any cause or last control. Actuarial time to progression and overall survival were determined by the method of Kaplan and Meier. Student-*t* test was applied to compare mean values. All *p*-values are two-tailed.

To determine the minimum size of the sample necessary to detect at least a 20% of activity, we followed the two-stage Phase II design of Gehan [14]. Once this activity was confirmed in the first 25 patients, it was decided to continue the study to further characterize the toxicity of the schedule in a multicenter setting.

## RESULTS

### Patient characteristics

From July 1997 to July 1999, 73 patients were included in the study. Two were considered ineligible (one with a low performance status and another exposed to prior chemotherapy). One patient was not evaluable for any analysis due to lack of data, 70 were assessable for toxicity and 64 for antitumor activity. Six patients were not valid for analysis of efficacy: 5 received only 1 cycle and one patient had a toxic death after the second cycle. Main characteristics of eligible patients are presented in Table 1. Retrospectively, a diagnosis of gastrointestinal stromal sarcoma tumor (GIST) was done in 8 patients. In 20 patients the primary tumor was the only site of disease and the lungs in 16. Sixteen patients had received radiotherapy before.

### Treatment

In total 277 cycles were delivered, with a median of 3 per patient (range 1–10). Six patients received only 1 cycle of therapy due to hematologic [1] or cardiac toxicity [1], treatment refusal [1], rapid progression [1] or a toxic death [2]. According to ifosfamide dose, 81 cycles were given at 10 g/m^2^, 130 at 12 g/m^2^, 33 at 13 g/m^2^, and 34 at 14 g/m^2^. In Table 2 we summarized the number of cycles given by ifosfamide dose level during the 6 cycles projected by protocol. Roughly, one third of cycles were given at 10, at 12, and at > 12 g/m^2^ IFOS during cycles 2 to 6. Five patients erroneously started therapy at 50 mg/m^2^ doxorubicin and 10 g/m^2^ ifosfamide: 1 refused further therapy, 2 had excessive toxicity for increasing dose, 1 continued at 10 g/m^2^ and 1 had the dose of ifosfamide increased to 12 g/m^2^ in subsequent cycles. Among the 65 patients who received the first cycle at 12 g/m^2^ ifosfamide, 22 had the dose increased to 13 g/m^2^ and 16 reduced to 10 g/m^2^ in the second cycle. The reasons to abandon the study during the first 6 cycles of therapy are summarized in Table 3. Ten out of 21 patients (48%) leaving the trial for toxicity were receiving IFOS at 10 g/m^2^ and 7 (33%) at 12 g/m^2^. Twenty patients completed 6 cycles, and 6 of them continued on therapy afterwards. Fifteen percent of the first 6 cycles were delayed, either due to toxicity (50%), patient request, or bed availability, median cycle interval being 28 days (range 26–35 days) with only 5% of cycles given on day 35. Mean dose received (± standard deviation) in those cycles was 50±1.58 mg/m^2^ for doxorubicin and 11.70±1.43 g/m^2^ for ifosfamide, and mean dose intensity was 11.98±0.70 mg/m^2^/week for DXR and 2.82±0.34 g/m^2^/week for IFOS.

### Hematologic toxicity

Sixty-nine patients and 254 cycles were assessable for hematologic toxicity. In Table 4 we present the maximum grade of this toxicity per patient during the whole treatment period. A grade 3 to 4 decrease in hemoglobin values was observed in 40% of patients (16% of cycles), and in platelet count in 43% of patients (19% of cycles), while 82% and 79% had grade 3 to 4 toxicity on leukocytes or granulocytes in 58% and 58% of cycles, respectively. The percentage of patients with grades 3 and 4 hematologic toxicity by dose of ifosfamide during the first 6 cycles of therapy is presented in Table 5. In those first 6 cycles, mean platelet values decreased with the number of cycles delivered (*P* < .0001). In patients with granulocytes < 1.000/mm^3^ (grade 3 to 4), the nadir occurred on day 12 (range 5–30), with mean granulocyte values of 0.22/mm^3^ (range 0–0.97). In 83% of cycles, recovery to minimum hematologic values required for therapy was already reached by day 21.

### Nonhematologic toxicity

The most frequent grade 3 to 4 side-effects were infection (46% of patients), vomiting (27%), asthenia (31%), anorexia (28%), and stomatitis (10%) (see Table 6). All episodes of neurocortical toxicity grade 3 consisted of visual hallucinations, while grade 1 to 2 episodes were usually characterized by a mild-to-moderate somnolence. Two patients referred asterixis with limitation to hold objects, and another two noted peripheral paresthesias. Serial LVEF determinations were available from 52 patients, and in 17 a mean decrease of 10.94 ± 4.14% was noted after therapy, with only 5 presenting values below 50% (range 41–47%). All 3 patients with serial measurements performed within the first cycle had a transient decrease in LVEF of 17%, 20%, and 7%, respectively, with one of them associated with symptomatic left cardiac failure that led to treatment interruption. Another patient who had received a cumulative DXR dose of 250 mg/m^2^ developed clinical cardiac failure 1 year after the end of protocol therapy, when an LVEF was 30%. Increased serum creatinine values observed in 9 patients were reversible in 6, 2 with grade 1 toxicity were not further evaluated, and one patient abandoned the study due to persistent creatinine clearance values < 60 mL/min concomitantly with a grade 1 increase in serum creatinine. No episodes of acute renal failure were observed.

During the study 38 patients (20% of cycles) had at least one episode of febrile neutropenia, requiring 50 hospital admissions for support. Although out-patient treatment through a central catheter was allowed, all patients but one received treatment as inpatients. If days of hospitalization due to both treatment and support measures are added, 75.4% of cycles required less than 7 days at hospital (median 5, range 3–6 days) and 24.6% more than 7 days (median 13, range 8–27 days). Red-packed cell transfusions were given to 41 patients in 29% of cycles, and 11 received platelet transfusions in 11 cycles. Sixteen patients in 23 cycles referred toxicity to GM-CSF that usually consisted of an itchy erythema plus mild swelling at the site of puncture (13 patients); 3 patients noted night sweats, fever, or a poor tolerance to GM-CSF administration. Two patients were shifted to G-CSF. All three episodes of toxic death were secondary to neutropenia plus unrecoverable sepsis in one patient coincidental with neurocortical and renal toxicity.

### Response to therapy and evolution

In 64 patients assessable for activity, the best response observed was a complete remission in 1 patient, a partial remission in 26, stable disease in 24, and progressive disease in 13, for an overall activity rate of 42% (95% CI, 30–54%). In 8 GIST patients, 1 partial remission, 2 stabilizations and 4 progressions were noted, with 1 being nonevaluable. If GIST patients are excluded, response rate was 45.6% (95% CI, 32–58%). Sensitive histotypes were leiomyosarcoma (8/14), angiosarcoma (2/4), neurogenic sarcoma (2/4), rhabdomyosarcoma (1/2), mixed mesodermal sarcoma (1/2), fibrosarcoma (2/6), synovial cell sarcoma (1/3), malignant fibrous histiocytoma (1/4) or liposarcoma (1/5) among others. Activity was noted on the primary tumor (44%), lung (48%), liver (33%), or other lesions (15%). Mean time (± SD) to achieve an objective remission was 2.8 ± 1.5 months (range 1.5–8 months). No correlation was observed between the mean dose of IFOS received and response. Mean duration of response was 7.87 months (95% CI, 5.88–9.85 months), and mean time to progression was 9 months (95% CI, 7–11 months).

The reason to abandon the study was excessive toxicity in 16 patients, treatment refusal in 2 (after 1 and 5 cycles, resp), salvage surgery in 6, disease progression in 19, and treatment completion in 24. Four patients finished protocol therapy after 4 cycles in the absence of toxicity or progression. Toxicity that led to treatment interruption consisted of hematologic depression associated to febrile neutropenia [11], poor general tolerance to therapy [4], or a persistent decrease in creatinine clearance (< 60 mL/min) in 1 patient. The occurrence of excessive toxicity was not related with sex, performance status, or site of disease, but those patients with severe toxicity tended to be older (mean ± SD, 54 ± 10 years versus 49 ± 9.7 years, *P* = .057). In all, 16 patients were submitted to rescue surgery (9 in partial remission and 7 with stable disease) and 11 apparently achieved a complete remission. Five of those patients also received adjuvant radiotherapy, and another 3 patients received radiotherapy upon completion of protocol therapy.

At the time of last analysis, 60 patients (86%) had died of disease, 3 had a toxic death, 2 were lost to follow-up (one of them without evidence of disease 6 years after completing therapy), and 5 patients were alive, 2 with active disease and 3 with no evidence of disease (two rescued with surgery and one with a durable complete remission after temozolomide). Median (± ds) overall survival for the whole series was 17 ± 2 months (95% CI, 13–22 months). No relation between survival and grade of hematologic toxicity was observed.

## DISCUSSION

In this study, ASTS patients were treated with IFOS at an initial dose of 12 g/m^2^ combined with DXR 50 mg/m^2^ every 4 weeks, with the dose of IFOS adjusted in the 10–14 g/m^2^ range according to the hematologic nadir. The dose of IFOS was increased in 34% of patients, and 30% had the dose reduced to 10 g/m^2^ in the second cycle. These proportions were maintained all along the study, so that approximately one third of patients each received 10, 12, and > 12 g/m^2^ IFOS (plus 50 mg/m^2^ DXR) during the first 6 cycles, pointing perhaps to interpatient differences in drug metabolism (Tables 2 and 5). A criticism to this trial may be the low dose of DXR administered, which led to a low dose intensity (12.5 mg/m^2^/week) compared with the 25 mg/m^2^/week of DXR given as a single agent at standard doses. As 83% of cycles had hematologic values suitable for repeating therapy on day 21, one can speculate whether this schedule would had been tolerated every 3 weeks. However, 26% of patients were taken off study due to excessive toxicity and three toxic deaths occurred, indicating that there is little margin for dose increase.

The three studies that explored a combination of IFOS 12 g/m^2^ plus DXR 60 mg/m^2^ repeated every 3 weeks in a small number of ASTS patients encountered excessive toxicity. In the first, only 85% of the planned dose for both agents was given [9]. The second was a Phase I trial testing two IFOS dose levels (10 and 12 g/m^2^) added to DXR (50–90 mg/m^2^). DXR 60 mg/m^2^ plus 12 g/m^2^ IFOS was the maximum tolerated dose due to the presence of neutropenia lasting more than 4 days and thrombocytopenia grade 4 in 3 out of 4 patients. However, when 10 g/m^2^ IFOS were combined with 60–90 mg/m^2^ DXR, the dose limiting toxicity was not reached [15]. In the third study, 8 out of 14 (57%) patients with metastatic disease treated at DXR 60 mg/m^2^ plus IFOS 12 g/m^2^ every 3 weeks discontinued therapy due to toxicity [16].

In this trial the dose of IFOS was adjusted in each patient in function of the toxicity. The dose limiting toxicity was febrile neutropenia, which affected 54% of patients, a figure equal to that observed in our Phase II trial with high-dose IFOS conducted in a similar patient population [12]. In spite of the prophylactic use of hematopoietic growth factors, grade 4 granulocytopenia occurs in 70–100% of patients treated with high-dose regimens, and febrile neutropenia is reported in 35–100% of patients exposed to DXR plus high-dose IFOS [8, 9, 15] or in 13–54% of those treated with combinations of EPI plus IFOS at 9–12 g/m^2^ [10, 17, 18], although septic deaths are seldom reported. This grade of toxicity must be accepted as inherent to any high-dose regimen applied to patients with ASTS. Grade 4 thrombocytopenia was detected in 19% of our patients. It was cumulative in nature, but did not lead to delays or interruptions of therapy. This incidence of grade 4 thrombocytopenia seems acceptable when compared with the 50% observed in trials which incorporated DXR at 75–90 mg/m^2^ to 10 g/m^2^ IFOS [8], or the 40–75% of the combination of DXR 60 mg/m^2^ plus IFOS 12 g/m^2^ [15, 16], what eventually limits the number of cycles that can be given at full doses. Other toxic effects noted are well known and were similar to those observed by others. In any case, these high-dose schedules are too toxic for routine use and should only be administered in experienced centers.

Due to the toxicity observed in this trial and in order to administer higher doses of DXR, we explored a sequential approach in a subsequent study, administering dose-dense DXR (3 cycles at 90 mg/m^2^ repeated every 2 weeks) followed by high-dose ifosfamide (3 cycles at 12.5 g/m^2^ every 3 weeks) [19]. In this trial, which included 57 patients, 7% of patients withdrew due to refusal or excessive toxicity, 24% had febrile neutropenia, 24% had thrombocytopenia grade 3 to 4, and 66% completed the 6 projected cycles of therapy. This regimen offered a better toxicity profile and compliance when compared with either the single agent IFOS or the presently reported study, with a similar antitumor activity (remission rate 38%; 95% CI, 25–51%), and it may provide a therapeutic option for ASTS patients. For this reason, it was selected for comparison with single agent DXR (75 mg/m^2^ every 3 weeks) in a Phase III study, presently under course. Prolongation of IFOS infusion offers another alternative, more cumbersome, to reduce the toxicity of DXR plus high-dose IFOS combinations. The study of the Italian Sarcoma Group showed that IFOS 13 g/m^2^ infused over 12 days and bolus DXR at 75 mg/m^2^ on day 8 could be combined every 4 weeks, with grade 4 neutropenia and thrombocytopenia observed, respectively, in 66% and 8% of patients [20].

Antitumor activity in ASTS (excluding GIST patients) was 45.6%, which is below the 54–69% of figures reported for other high-dose regimens tested [8, 10, 15, 17, 18, 20], with the exception of the 25% reported in 12 metastatic patients [16]. Perhaps the low DXR dose administered influenced our results, although this figure coincides with that of other studies from our Group [12, 19], and no definitive conclusions can be drawn from small trials conducted in such a heterogeneous group of tumors. However, the results of most studies are consistent, and suggest that high-dose regimens may have a higher efficacy than standard-dose schedules, an impression that should be tested in adequately sized randomized trials. Salvage surgery was possible in 25% of evaluable patients, and 56% of them had obtained a partial remission during protocol therapy. Rescue surgery is performed in about 30% of ASTS patients included in high-dose studies [10, 17, 18], being difficult to deduce to what extent chemotherapy has facilitated surgery. For this reason, resectability of lesions should be established beforehand through clear definitions included among the selection criteria. This aspect, addressed in a recent paper [21], could be considered a stratification criterion in future studies.

From the therapeutic results obtained in some solid tumors, it has been proposed that hematologic toxicity is a better parameter than dose intensity to measure dose adequacy, and that outcome is related to the grade of that toxicity [22]. According to this, the doses of any chemotherapeutic regimen should be adjusted to cause at least moderate hematologic toxicity to every patient to compensate for differences in tolerance [23]. In ASTS patients, it is difficult to test that proposal due to the very heterogeneous nature of the tumors grouped under the term sarcoma. With this contention, if toxicity is related to outcome, then the regimen we tested would have reached a limit, as well as other high-dose regimens combining IFOS and an anthracycline, that will unlikely be improved by pushing further the doses delivered. New agents would be necessary to improve the poor therapeutic results obtained in ASTS patients, and the differences in biology and in sensitivity between the different sarcoma subtypes should be exploited.

## Figures and Tables

**Table 1:** Patient characteristics.

Included	73
Eligible	71
Male/female	29/42
Median age (range)	55 (22–65)
	
Performance status:	
0	24
	
1	34
2	13
	
Histology:	
Leiomyosarcoma	16
	
GIST	8
Fibrosarcoma	6
Liposarcoma	6
Malignant fibrous histiocytoma	5
Angiosarcoma	4
Neurosarcoma	4
Synovial cell sarcoma	3
Rhabdomyosarcoma	2
Mixed mesodermal sarcoma	2
Other	22
	
Grade of malignancy:	
1	14
	
2	22
3	35
	
Sites of disease:	
Primary/local recurrence	41
	
Lung	29
Liver	10
Other	13

**Table 2:** Number of patients and dose of ifosfamide during the first 6 cycles of treatment.

	Dose of ifosfamide			

	10 g/m^2^	12 g/m^2^	13 g/m^2^	14 g/m^2^	Total

Cycle number					(patients)

1	5^(a)^ (0.07)	65 (0.93)	—	—	70
2	19 (0.30)	23 (0.36)	22 (0.34)	—	64
3	18 (0.37)	17 (0.35)	4 (0.08)	10 (0.20)	49
4	15 (0.40)	10 (0.27)	4 (0.11)	8 (0.22)	37
5	9 (0.37)	6 (0.25)	3 (0.13)	6 (0.25)	24
6	8 (0.40)	6 (0.30)	2 (0.10)	4 (0.20)	20

^(a)^Number of patients (%).

**Table 3:** Reasons to abandon the study during the first 6 cycles of treatment.

	Toxicity^(a)^	Progression	Additional	End of	Total
			therapy^(b)^	therapy	

Cycle number

1	5^(c)^	1	—	—	6
2	8	7	—	—	15
3	5	6	1	—	12
4	2	2	5	4	13
5	1	2	1	—	4
6	—	—	8	6	14

^(a)^Toxicity includes excessive toxicity, patient refusals, and toxic deaths.

^(b)^Surgery, radiotherapy or both.

^(c)^Number of patients.

**Table 4:** Maximum hematologic toxicity (% of patients).

	Grade CTC				

	0	1	2	3	4

Hemoglobin	1	6	53	31	9
Leukocytes	8	—	10	22	60
Granulocytes	12	3	6	12	67
Platelets	15	29	13	24	19

**Table 5:** Grades 3 and 4 hematologic toxicity by ifosfamide dose during the first 6 cycles (% of patients).

	Dose of ifosfamide			

	10 g/m^2^	12 g/m^2^	13 g/m^2^	14 g/m^2^

Hemoglobin	21/7	20/3	9/4.5	36/9
Leukocytes	18/57	19/42	41/23	27/45
Granulocytes	0/71	19/46	9/45	9/63
Platelets	46/11	14/9	9/9	0/36

**Table 6:** Nonhematologic toxicity (% of patients).

	Grade				

	0	1	2	3	4

Nausea	10.1	20.3	48	20.3	1.4
Vomiting	18.6	17.1	37.1	24.3	2.9
Diarrhea	81.2	11.6	5.8	1.4	—
Stomatitis	54.3	16	20	8.6	1.4
Asthenia	7.4	17.6	44.1	25	6
Anorexia	13.2	25	34	23.5	4.4
Cardiotoxicity	81.2	11.6	3	4.3	—
Neurocortical	55	24.6	7.2	13	—
Cutaneous	88	6	6	—	—
Alergia	93	6	1	—	—
Fever	38.6	7	38.6	7.1	8.6
Infection	45	3	6	36	10
Creatinine	86	11	3	—	—
Hematuria	48	40	8	4	—
